# Dengue Mimickers: Which Clinical Conditions Can Resemble Dengue Fever?

**DOI:** 10.1590/0037-8682-0334-2024

**Published:** 2024-12-16

**Authors:** Luis Arthur Brasil Gadelha Farias, Lourrany Borges Costa, Pedro Pinheiro de Negreiros Bessa, Glaura Fernandes Teixeira de Alcântara, Jobson Lopes de Oliveira, Thalita do Nascimento Silva, Giuliana de Fátima Lima Morais, Lauro Vieira Perdigão, Luciano Pamplona Góes Cavalcanti

**Affiliations:** 1 Universidade de São Paulo, Departamento de Doenças Infecciosas do Hospital das Clínicas, Laboratório de Investigação Médica - LIM 49, São Paulo, SP, Brasil.; 2 Hospital São José de Doenças Infecciosas, Fortaleza, CE, Brasil.; 3 Centro Universitário Christus (Unichristus), Faculdade de Medicina, Fortaleza, CE, Brasil.; 4 Universidade Federal do Ceará, Departamento de Medicina Clínica, Fortaleza, CE, Brasil.; 5 Universidade de Fortaleza, Fortaleza, CE, Brasil.; 6 Hospital Geral Dr. César Cals, Departamento de Reumatologia, Fortaleza, CE, Brasil.; 7 Universidade Federal do Ceará, Faculdade de Medicina, Fortaleza, CE, Brasil.

**Keywords:** Dengue, Dengue Fever, Dengue Virus, Internal Medicine, Clinical Medicine

## Abstract

Epidemics and outbreaks caused by the dengue virus pose risks to populations and have high mortality rates, causing burdens and economic costs worldwide. Brazil recently experienced an explosive increase in the number of dengue cases and fatalities. Dengue is an acute febrile illness that can progress to severe forms. It affects more than 100 countries, presenting ongoing challenges in Brazil and globally since its identification. Other conditions may be overlooked or mistaken for dengue. The most important differential diagnoses are other infectious diseases and rheumatological, hematological, gastroenterological, and neurological disorders. In this article, we discuss the primary differential diagnoses of dengue and offer a literature review highlighting the key clinical differences among clinicians. This review emphasizes the critical importance of differentiating dengue fever from infectious diseases such as meningococcemia and malaria and autoimmune and rheumatological conditions such as systemic lupus erythematosus to ensure timely and appropriate management.

## INTRODUCTION

Dengue (DEN) is a viral disease transmitted by *Aedes aegypti* mosquitoes*.* DEN has been a challenge in Brazil and worldwide since its discovery in the late 18th century^1^. The DEN virus is endemic to over 100 countries, with outbreaks associated with four serotypes (DEN-1, DEN-2, DEN-3, and DEN-4)^2^. Epidemics and outbreaks caused by DEN pose risks to populations and have high mortality rates, causing burden and economic costs worldwide for decades^3^. In 2024, Brazil will experience an early and explosive rise in DEN cases, with more than six million cases reported in the first six months of the year and multiple states registering an unprecedented increase compared to their historical series^4^. To the best of our knowledge, this is the worst DEN epidemic in Brazil's history, reinforcing the need for and stigma surrounding neglected tropical diseases (NTDs) that require further study and the implementation of public health measures for prevention^3^ ^,^ ^4^. DEN has been a growing public health concern in Brazil for decades, which has motivated research, vaccine development, and vector control measures^4^ ^,^ ^5^. 

DEN is typically characterized by fever, arthralgia, myalgia, rash, headache, and retroocular pain, which may evolve into mild to severe hemorrhagic complications^4^. Although DEN's clinical presentation of DEN is well known and its characteristics are well established, many conditions may mimic DEN, generating doubts about its diagnosis and delaying correct disease management. Differential diagnoses have implicated numerous diseases, including rheumatological, hematological, gastroenterological, and neurological disorders. Given the high possibility of DEN during the current outbreak, these cases were highly suspected. However, delays or misdiagnosis can lead to inappropriate treatment and increased morbidity and mortality. Understanding the differential diagnoses and their particularities is crucial for clinicians to avoid errors and perform appropriate case management. 

Herein, we describe the main differential diagnoses of DEN and conduct a brief literature review. The differential diagnoses and similarities with DEN were based on previous expert experience and cases encountered during clinical practice.

## METHODS

Seven clinical experts conducted this review: five infectious disease specialists, two rheumatologists, one epidemiologist, and one family and community health specialist. We compiled information from previously published, peer-reviewed studies to describe the main differential diagnoses that may mimic DEN. Studies were included based on their clinical relevance at the discretion of the authors. Systematic and comprehensive reviews, meta-analyses, and observational studies were included. Case reports, letters, and series were included to represent important differential diagnosis^6^. The LILACS, PubMed, and Google Scholar databases were searched. We categorized the data into rheumatological, hematological, infectious, gastrointestinal, and neurological conditions, based on the predominant medical area of each condition. After obtaining informed consent, the authors provided personal images to illustrate the diagnoses. The primary focus is to highlight the key clinical differences between differential diagnoses and DEN and alert physicians to the necessity of ruling out other diagnoses.

## INFECTIOUS DISEASES

**FIGURE 1:**
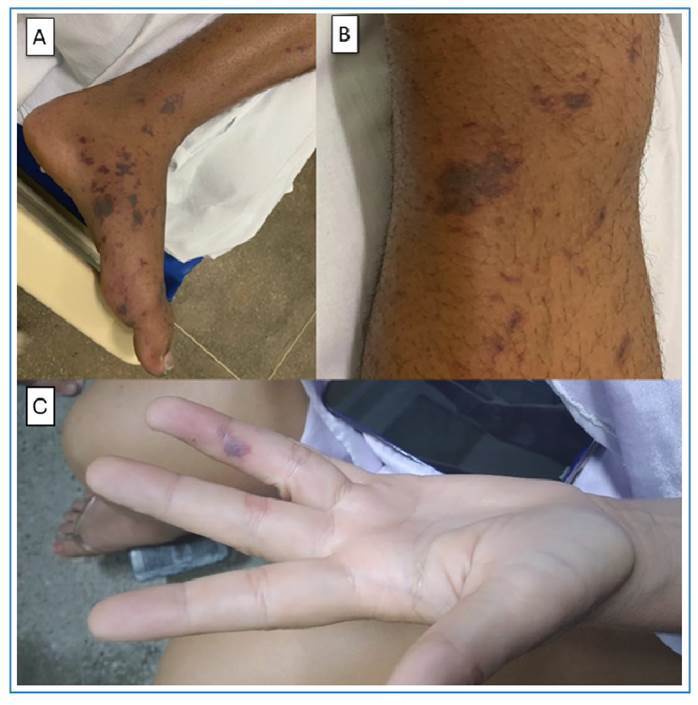
**A.** Meningococcal purpura fulminans lesions on the feet. **B.** Classical meningococcal skin lesions with a violaceous hue and petechiae on the lower limbs. **C and D.** Petechial lesions on the hands and feet in a 19-year-old patient with meningococcemia, initially misdiagnosed as dengue (DEN). ***Source: Personal archive of Dr. Pedro Bessa.***

*Meningococcemia:* Meningococcemia is a severe and often fatal bacterial infection caused by *Neisseria meningitidis* ^7^. Although DEN is a viral disease, the initial symptoms are indistinguishable^8^. Fever and rash are features shared by both conditions. It was initially difficult to distinguish between DEN and meningococcemia^8^. As meningococcemia progresses, more distinguishable findings such as violaceous or necrotic skin lesions may appear (Figure 1). Meningococcemia with meningitis at onset can mimic the headache observed in DEN and the initial petechial purpuric lesions before they coalesce. Intense headaches were observed in both conditions. Headache presented in DEN is usually holocranial and may be accompanied by retro-orbital pain. Indeed, fever with rash can signal a life-threatening infection and imminent clinical decompensation, with meningococcemia as a potential cause. Another study in children showed that meningococcemia presents with symptoms of neck stiffness, rash, photophobia, confusion, or leg pain, which may be confused with the fever, headache, and rash usually seen in most DEN cases^9^. A total blood count should help differentiate bacterial infections, with leukocytosis being a notable finding. Counterintuitively, the absence of leukocytosis does not rule out meningococcemia and may be related to the poor prognosis in these patients^8^ ^,^ ^10^. Inflammatory markers such as C-reactive protein (CRP) and erythrocyte sedimentation rate (ESR) are typically elevated in bacterial infections, but can be nonspecific. Souza et al. found that the ESR was below the reference value in most patients with DEN, especially in hemorrhagic forms^11^. In 2023, during a DEN outbreak in Rio de Janeiro, Filippis et al. described a case of fever and rash caused by coinfection with *Neisseria meningitidis* and DEN, which had a fatal outcome^10^. This case underscores the need to differentiate between the two diseases and remain vigilant regarding the possibility of coinfection during epidemic periods^10^. If there is clinical uncertainty regarding whether the condition is DEN or meningococcemia, ceftriaxone 2 g per day should be immediately initiated to prevent a fatal outcome due to bacterial infection, especially in low-income medical contexts. 

*Malaria:* Malaria and DEN are acute febrile illnesses with many similarities^12^. Mosquitoes transmit both diseases, although the *Anopheles* genus transmits malaria, whereas *Aedes* transmits DEN. These diseases share a similar epidemiology, affecting subtropical and tropical countries, particularly in the Southeast Asia and Asia-Pacific regions^13^ ^-^ ^15^. Clinically, differentiating between these two conditions may be challenging and even more difficult outside the Amazon regions^13^ ^,^ ^14^. Malaria is an acute febrile illness characterized by a low platelet count and anemia, similar to DEN. However, the clinical characteristics of the fever may differ from those of DEN. The clinical predictors of malaria include travel history, particularly in the last 30 days, chills, and sweating^13^. Malaria typically presents with fever episodes characterized by tertiary or quartan fever, depending on the *Plasmodium* sp. However, this feature may sometimes be present or clearly described in the clinical history^13^ ^,^ ^14^. Patients from the northern region of Brazil may present with co-infection of malaria and DEN^16^. Regardless of whether Malaria or DEN is suspected, careful monitoring of bleeding and hepatic complications is recommended, which may result in a higher chance of severity^12^ ^,^ ^16^ ^,^ ^17^. Thick blood smear examination is essential because of its easy implementation and rapid visualization of *Plasmodium* spp., especially in endemic areas^18^. During the acute phase, both diseases may be diagnosed by qPCR focusing on suspected pathogens, which may be helpful^12^ ^,^ ^14^ ^,^ ^16^ ^,^ ^18^. The NS1 antigen test is another alternative for DEN diagnosis and is frequently positive during the first five days of acute onset^1^ ^-^ ^3^ ^,^ ^12^.

***COVID-19 and Influenza:*** Differentiating DEN from respiratory infections, such as COVID-19 and Influenza, has been challenging since the beginning of the COVID-19 pandemic in 2020^19^. This challenge remains because of many overlapping signs or symptoms, which provoke a major diagnostic challenge for healthcare providers in different clinical contexts^20^ ^,^ ^21^. Several clinical studies have been conducted in Puerto Rico, Singapore, and China^19^ ^-^ ^21^. Thein et al. enrolled patients with COVID-19 (n=126), influenza (n=171), and DEN (n=180) to identify the clinical predictors for differentiating these conditions and understand the main clinical differences^19^. In their research, dyspnea was the strongest predictor of COVID-19 and influenza, followed by diarrhea. A higher lymphocyte count predicted COVID-19 than influenza and DENF, while headache, joint pain, skin rash, and vomiting/nausea indicated DEN^19^. Another study conducted by Gerardin et al. at Saint-Pierre Hospital in Reunion Island involved the development of two scores, COVIDENGUE, by analyzing and identifying the predictors of DEN, COVID-19, and other febrile illnesses. The results showed that anosmia was a strong predictor of COVID-19, whereas body aches were a strong predictor of DEN^20^. DEN and COVID-19 have distinct clinical features. Symptoms of DEN include retro-orbital eye pain, photophobia, bleeding, and petechiae^1^ ^-^ ^3^ ^,^ ^12^. COVID-19 can cause loss of smell, leading to taste disturbances, erythematous rashes, urticaria, thrombotic complications, and consumptive coagulopathy^22^. Both conditions present with myositis as a clinical feature characterized by the presence of myalgia and elevated creatine kinase levels. In contrast to DEN, influenza-induced myositis is a marker of severity^23^.

During the COVID-19 pandemic, some countries have experienced a decrease in DEN cases due to isolation measures. However, the misdiagnosis of DEN and COVID-19 occurs because of similar clinical symptoms, including fever, dyspnea, headache, cough, and skin manifestations. During outbreaks of both diseases, the likelihood of co-infection increases, with false-positive serological tests being common, suggesting cross-reactivity. Thus, for an accurate diagnosis of DEN, it is necessary to use NS1 antigen-based rapid diagnostic tests. Both diseases cause severe complications by triggering cytokine storm^22^. During influenza outbreaks, despite the respiratory symptoms of influenza, DEN may also be misdiagnosed as influenza and vice-versa^24^ ^,^ ^25^. The seasonality of DEN and influenza can overlap or occur during the same period, complicating their differentiation^20^ ^,^ ^21^ ^,^ ^24^. Table 1 summarizes the main clinical and laboratory differences between DEN, Influenza, and COVID-19.

**TABLE 1:** Signs and symptoms of respiratory conditions that may resemble DEN.

Signs and Symptoms	DEN^1^ ^-^ ^3^ ^,^ ^11^	Influenza^20^ ^,^ ^24^	COVID-19^20^ ^,^ ^21^ ^,^ ^23^
**Fever**	Above 38ºC	Above 38ºC	Above 38ºC
**Runny nose**	Absent	Present	Present
**Dyspnea**	Absent or during the second DEN phase	More frequent	More frequent
**Anosmia**	Absent	Absent	Present
**Rash**	Severe	Mild or Absent	Moderate to Severe
**Lymphopenia**	Present	Mild or Absent	Present
**Diarrhea**	Mild or Absent	Present	Present

***Chikungunya, Zika and Yellow Fever:*** Similarities between the DEN virus and other arboviruses have been well-documented^26^. Multiple epidemics of similar diseases have intrigued scientists and spurred the discovery of other arboviruses throughout history^26^ ^,^ ^27^. In 1953, an acute febrile illness resembling DEN was reported in the Tanganyika region of Tanzania, providing evidence of a new virus known as the chikungunya virus (CHIKV)^27^. The similarities between CHIKV and DEN are evident; both diseases can present with high fever, arthralgia, myalgia, and rash. In Brazil, CHIKV was introduced in Amapá, at the border with French Guiana (Asian Lineage), as well as in Bahia, in the Northeast (East/Central/South African genotype-ECSA lineage) in 2014, when it was initially mistaken for the DEN virus. The main clinical difference between the two conditions is the ability of CHIKV to cause more intense polyarthralgia and arthritis as well as to present a post-acute and chronic phase, which can lead to lasting sequelae and morbidity^28^. In addition, a recent study from Puerto Rico that compared DEN with other arboviruses found that CHIKV may present with an early rash and more musculoarticular symptoms than DEN^29^. In the same study, patients with CHIKV were less likely to present with thrombocytopenia, signs of poor circulation, diarrhea, headache, and cough^29^. Currently, CHIKV continues to circulate and cause cases in the country, often being differentiated from DEN through serology, which shows about a 6% cross-reactivity rate^30^.

In Brazil, the introduction of the Zika virus (ZIKV) was similar to that of CHIKV. In 2014, some areas of Pernambuco State reported an uncommon illness characterized by low or absent fever, rash, pruritus, and conjunctivitis, resembling DEN^26^ ^,^ ^31^ ^,^ ^32^. The clinical picture of ZIKV became well-known after its epidemic in Brazil, along with complications related to microcephaly and Guillain-Barré syndrome. An important feature is the low or absent fever observed in ZIKV, in contrast to the DEN virus^26^ ^,^ ^31^ ^,^ ^32^. Serological diagnosis is difficult because ZIKV and DEN are flaviviruses and serological tests can show high cross-reactivity rates^32^. During the acute phase, qPCR is recommended for both viruses is recommended^33^. Conjunctivitis without purulent secretion has been described in ZIKV infection^31^ ^-^ ^33^. This feature is not commonly observed in DEN but is possible and has been previously described^34^.

Yellow fever is caused by viruses belonging to the Flaviviridae family, specifically the Flavivirus genus, such as DEN virus and ZIKV^35^. DEN and yellow fever virus (YFV) occur in overlapping geographical areas. Nevertheless, one hypothesis suggests that one virus can alter another 's epidemiology through competition, a phenomenon observed in certain Asian countries. Counterintuitively, it is hypothesized that a mosquito infected with the DEN virus is incapable of being infected by YFV and vice versa, and a person who has recovered from DEN is unable to contract yellow fever and vice versa. Thus, diseases with a lower basic reproduction number are excluded^36^. This may account for the infrequent occurrence of DEN and YFV infections. Therefore, yellow fever is a significant differential diagnosis, exhibiting distinct clinical variations, including jaundice, and more prevalent liver damage^1^ ^-^ ^3^ ^,^ ^12^ ^,^ ^35^ ^,^ ^37^. Both diseases may result in bleeding; however, hepatic impairment is more common in yellow fever^26^ ^,^ ^35^ ^,^ ^37^.

*Ae. aegypti* mosquitoes transmit CHIKV, ZIKV, DEN, and YFV. *Ae. aegypti* and *Ae. albopictus* is known to transmit all four DEN serotypes, YFV, CHIKV, and ZIKAV, and has been suggested to be a potential vector of the Venezuelan equine encephalitis virus^38^ ^,^ ^39^. *Ae. albopictus* seems to be a more effective vector for CHIKV, causing disease within a few days of ingesting infected blood^38^. *Ae. albopictus* presented better adaptability and improved transmission of CHIKV than other arboviruses. It has been proven that both vectors, when co-infected with DEN and CHIKV, can replicate and disseminate both viruses independently^38^. Studies have shown the possibility of simultaneous transmission of more than one virus in a single bite^38^. These viruses cocirculate in Brazil, which may complicate the diagnosis of DEN and highlight the need to suspect coinfections^38^ ^,^ ^39^. Other countries, such as Colombia and Venezuela, have demonstrated the simultaneous co-circulation of DEN, CHIKV, ZIKV, and their co-infections, which are challenging to differentiate clinically and may require molecular diagnosis^26^ ^,^ ^32^ ^,^ ^38^ ^,^ ^39^. Table 2 summarizes the differences between DEN and the most common arboviruses in Brazil.

**TABLE 2:** Summary of Differences Between DEN and the Most Common Arboviruses from Brazil.

Vírus	Signs and Symptoms	Complications	Prevalence in Brazil	Diagnostic Challenges
**DENV**	High fever, headache, pain behind the eyes, muscle and joint pains, rash^1^ ^-^ ^3^.	Severe dengue can cause hemorrhagic fever, shock, and organ failure.	It is highly prevalent, especially during the rainy season.	Cross-reactivity in serological tests with other flaviviruses^30^.
**CHIKV**	High fever, severe joint pain, muscle pain, headache, rash^27^ ^-^ ^30^.	Chronic joint pain, which can persist for months or years.	Significant outbreaks have occurred and may have high mortality rates^134^.	Distinction from DENV based on joint pain severity and duration. Cross-reactivity is not common but possible^30^.
**ZIKV**	Low or no fever, rash, conjunctivitis, muscle and joint pain, malaise^32^ ^-^ ^35^.	It is linked to microcephaly in newborns and Guillain-Barré syndrome in adults.	It emerged significantly in 2014 and is still being transmitted.	Cross-reactivity with other flaviviruses, especially DENV^30^.
**YFV**	Fever, chills, severe headache, back pain, general body aches, nausea and vomiting^35^ ^-^ ^37^.	Severe cases can cause liver damage, jaundice, and bleeding.	Endemic in certain regions; vaccination effectively controls spread.	Cross-reactivity with other flaviviruses, especially DENV^36^.
**OROV**	Fever, headache, myalgia, arthralgia, skin rashes, malaise, nausea and vomiting ^40^ ^,^ ^43^ ^,^ ^46^ ^,^ ^47^. After 1 to 2 weeks, there may be a recurrence of fever and headache^40^ ^,^ ^43^ ^,^ ^46^ ^,^ ^47^.	May evolve with hemorrhagic symptoms and neurological complications similar to DEN^43^ ^,^ ^46^ ^,^ ^47^.	In 2024, the Amazon region, considered endemic, accounted for 79.6% of the cases reported in the country. Autochthonous transmission in non-Amazonian states was recorded in BA, CE, MS, PR, ES, MA, MG, PE, MT, RJ, SC, and PI^47^.	Diagnosis can be challenging due to clinical overlap with other arboviruses and limited awareness.

**DENV:** Dengue virus; **ZIKV:** Zika virus; **CHIKV:** Chikungunya virus; **YFV:** Yellow Fever. **OROV:** Oropouche virus.

***Other Arboviruses:*** Brazil has more than 200 described arboviruses, some of which have the potential to cause diseases in humans that closely resemble DEN clinically^40^. Most arboviruses that cause human diseases present as acute febrile illnesses and may initially be misdiagnosed as DEN^1^ ^-^ ^3^ ^,^ ^12^ ^,^ ^13^ ^,^ ^40^ ^,^ ^41^. Examples of flaviviruses that circulate in Brazil and may cause diseases in humans, potentially causing serological cross-reactions due to their similarity to DEN, include Bussuquara, Cacipacoré, Iguape, Ilhéus, Rocio, Saint Louis encephalitis, and yellow fever^41^. A noteworthy challenge is that other flaviviruses, such as YFV, Rocio, Cacipacoré, and Zika, may exhibit cross-reactivity with DEN, complicating diagnosis and highlighting the need for molecular methods based on RT-PCR^30^ ^,^ ^36^. Other genera, such as Alphavirus and Orthobunyavirus, can also cause human diseases and mimic DEN, as exemplified by Oropouche and Mayaro viruses^40^ ^,^ ^41^. Knowledge and awareness of the cocirculation of these viruses is fundamental, and the availability of molecular methods is necessary to investigate their differential diagnoses. Herein, we describe three other arboviruses similar to DEN, outlining their particularities and current epidemiological importance, mainly in endemic areas, such as Brazil^1^ ^-^ ^3^ ^,^ ^12^ ^,^ ^41^.

***Mayaro virus:*** Mayaro virus (MAYV) is responsible for outbreaks of acute febrile illness in the Amazon region and the Central Plateau of Brazil, as well as in other South American countries (Peru, Bolivia, and Venezuela)^41^. MAYV strikes people working or living in the Amazon Forest and should be considered as a differential diagnosis. Physicians must be aware of its circulation, and contact with virological epidemiological surveillance is most important^41^. A remarkable finding that may differentiate it from DEN is the presence of intense polyarthralgia and/or polyarthritis; however, distinguishing it is difficult to distinguish from CHIKV^42^. Thus, Mayaro causes high fever, eye pain, and rashes, which may be indistinguishable from DEN^42^.

***Oropouche virus:*** Oropouche virus (OROV) is an arbovirus that circulates in South and Central America^41^ ^,^ ^43^. Epidemics in the Americas, including Brazil, have been described over the last 60 years^40^ ^,^ ^41^ ^,^ ^43^ ^,^ ^44^. The disease was first identified in Trinidad and Tobago in the 1950s, and OROV was subsequently isolated from Brazil in 1960^43^. Increasing incidences of OROV and MAYV have been reported in Acre state, Brazil^43^. Brazil is experiencing a 448.86% increase in OROV cases, affecting all macroregions, highlighting the need to recognize this arbovirus as a differential diagnosis^45^ ^-^ ^47^. It manifests as an acute febrile disease during the rainy season^40^ ^,^ ^43^. The general clinical presentation of DEN may resemble that of DEN, which can lead to confusion. OROV fever is a self-limiting, DEN-like acute febrile illness that lasts for 2-7 days. It is associated with various symptoms including fever, chills, headache, myalgia, arthralgia, malaise, dizziness, nausea, vomiting, photophobia, and retro-ocular pain^40^ ^,^ ^43^ ^,^ ^44^. A skin rash, which more commonly appears on the trunk and arms, is also possible, but differs from that typically seen with DEN. Hemorrhagic signs, such as spontaneous bleeding, petechiae, epistaxis, and gingival bleeding, were similar to those observed in DEN. Additionally, central nervous system symptoms, such as aseptic meningitis or meningoencephalitis, often seen in severe cases of DEN, have also been described in infections caused by OROV^40^ ^,^ ^43^ ^-^ ^47^. 

***West Nile virus:*** The West Nile virus (WNV) is an RNA virus belonging to the family Flaviviridae, genus Flavivirus, and the DEN virus^42^ ^,^ ^48^. Most (80%) of WNV infections are asymptomatic, but the few symptomatic infections are usually mild and self-limiting febrile illnesses^48^. Most symptomatic patients exhibit fever, sometimes associated with headache, myalgia, nausea, vomiting, and chills, which are the classic symptoms of DEN^1^ ^-^ ^3^ ^,^ ^48^. Understanding West Nile Fever involves recognizing that its main complications may affect the central nervous system, occurring in approximately 1% of cases. These complications can manifest as meningitis or encephalitis, similar to the neurological complications found in DEN. If there is suspicion of a neuro arbovirus, WNV should be included among the differential diagnoses and the more commonly known arboviruses^48^ ^,^ ^49^. Paddock et al. described a case of WNV infection in a 59-year-old man from Florida, United States, which led to a fatal outcome due to extensive hemorrhage, similar to that observed in DEN. In the present case, DEN, Rocky Mountain Spotted Fever, and Yellow Fever were among the differential diagnoses^50^.

***Leptospirosis:*** Leptospirosis and DEN cause concern in tropical countries during the rainy season in tropical countries^51^. Leptospirosis is a zoonosis with clinical protean manifestations, caused by pathogenic spirochetes belonging to the genus *Leptospira* ^51^. Outbreaks and epidemics of both diseases can occur concurrently during the rainy season in certain countries, posing challenges in identifying the specific disease responsible for them^52^. During these epidemics, concerns have been raised about the risk of confusion between leptospirosis and DEN, because the clinical biological presentations during the acute phase of both diseases are often similar^1^ ^-^ ^3^ ^,^ ^12^ ^,^ ^51^. Confusion between these diseases may delay antibiotic treatment, leading to increased mortality in leptospirosis patients^51^ ^-^ ^53^. 

Leptospirosis may present with many overlapping symptoms such as DEN^51^. In contrast, leptospirosis may present with distinct features not typically observed in DEN, such as calf pain, myalgia, conjunctival suffusion, jaundice, and conjunctival hemorrhage. However, in rare cases, myalgia and severe hemorrhage affecting the eye can mimic leptospirosis^51^ ^-^ ^53^. AKI is usually a part of Weil’s disease, composed of kidney and hepatic dysfunction and hemorrhages, and may also be present in DEN, but in a few cases^51^. The primary manifestations of AKI in patients with DEN include non-oliguric renal failure associated with hypokalemia and hyponatremia due to increased fluid loss. Leptospirosis could directly affect electrolyte transport mechanisms, leading to disturbances in sodium (Na⁺), chloride (Cl⁻), and potassium (K⁺) levels by inhibiting the Na⁺/K⁺/Cl⁻ cotransporter. Urea nitrogen levels generally remained at 100 mg/dL^51^. In leptospirosis, AKI develops because of dehydration, interstitial nephritis, and possibly immune complex-mediated glomerulonephritis^51^. AKI is a potential complication of severe DEN and is typically associated with hypotension, rhabdomyolysis, and hemolysis. AKI occasionally complicates severe DEN infections and results in high mortality rates^1^ ^-^ ^3^.

During the acute phase, common laboratory findings include neutrophilia; however, the total white blood cell (WBC) count may be normal, decreased, or elevated. Thrombocytopenia and anemia are less frequently observed; however, pancytopenia has been documented as an initial presentation in some case reports^51^ ^-^ ^53^. In a retrospective study from the Reunion Islands, Maillard et al. identified an increased CRP level (>50 mg/L) as a biomarker to diagnose leptospirosis and aid the decision-making process for hospital surveillance and/or a potential antibiotic treatment regimen^54^. 

***Mono-like Infections:*** Mono-like infections such as Epstein Barr virus (EBV), Cytomegalovirus (CMV), human immunodeficiency viruses (HIV), and Toxoplasma infections are prevalent in daily clinical practice and often simulate DEN. A study during a DEN outbreak in São Paulo, Brazil, after excluding individuals with a known HIV diagnosis, revealed an HIV-1 prevalence of 0.73% was found^55^. Other studies involving patients returning from tropical countries that evaluated mono-like infections found a prevalence of common agents, including CMV, *T. gondii*, EBV, and HIV primary infections, less commonly with DEN (only four patients). Despite the reduced number of patients with DEN, this diagnosis should also be ruled out as it mimics or presents with mono-like infections^56^. 

Mononucleosis is often mistaken for pharyngotonsillitis because of the high fever and similar appearance of purulent lesions, frequently leading to unnecessary antibiotic use^57^. A diagnostic clue to distinguish mononucleosis is the occurrence of a rash after amoxicillin administration, which is associated with the condition and differs clinically from the rash in DEN^1^ ^-^ ^3^ ^,^ ^30^ ^,^ ^57^. In mononucleosis, this rash is usually related to the antibiotics administered, whereas in DEN, the rash typically appears around the fourth day of illness, during the defervescence period, and is not associated with antibiotic use^1^ ^-^ ^3^ ^,^ ^30^. Mono-like infections may present with laboratory abnormalities, such as thrombocytopenia and elevated C-reactive protein levels, often leading to confusion. The similarities between Epstein-Barr virus (EBV) and DEN symptoms frequently lead to serological tests differentiating between diagnoses. Boyd et al. described a 46-year-old traveler who developed fever, chills, headaches, myalgia, fatigue, and photophobia one day after returning from the Philippines^58^. The patient demonstrated mild transaminitis and significant thrombocytopenia (12,000 cells/μL), as an acute DEN infection. Initial evaluation revealed a positive heterophilic antibody test for Epstein-Barr virus (EBV). Fortunately, due to the absence of classic EBV presentation and travel history, a DEN test by direct fluorescence IgM and IgG was positive. He did not have a positive result for the EBV DNA polymerase chain reaction or immunoglobulin M, the IgM by the viral capsid antigen. This suggests that there may have been a false positive result between EBV and DEN. Another similar case of a patient with a clinical picture resembling typhoid fever or EBV infection had positive titers for EBV and DEN, revealing the possibility of a cross-reaction^59^. Serological cross-reactivity between EBV and DEN seems rare but needs to be considered mainly in DEN-endemic settings^58^.

***Other Infectious Diseases:*** In the pediatric age group, exanthematous diseases such as Roseola Infantum or Parvovirus B19 infections can also mimic DEN^39^ ^,^ ^60^ ^,^ ^61^. Despite the characteristic rash, more common diseases such as Measles and Rubella can be misdiagnosed as DEN. DEN does not present with posterior auricular or suboccipital lymphadenopathy or a cephalocaudal rash of the rubella^59^ ^,^ ^60^. Conjunctivitis, headache, and polyarthritis in measles and rubella may confuse pediatricians^60^ ^,^ ^61^. Exanthema subitum is also described as an exanthematous disease that needs to be differentiated from DEN in children and adults. Human herpes virus 6 (HHV-6) causes exanthema subitum. It usually does not present with a high fever, although the exanthem may be indistinguishable from DEN^1^ ^-^ ^3^ ^,^ ^30^ ^,^ ^39^ ^,^ ^60^ ^,^ ^61^. 

**FIGURE 2:**
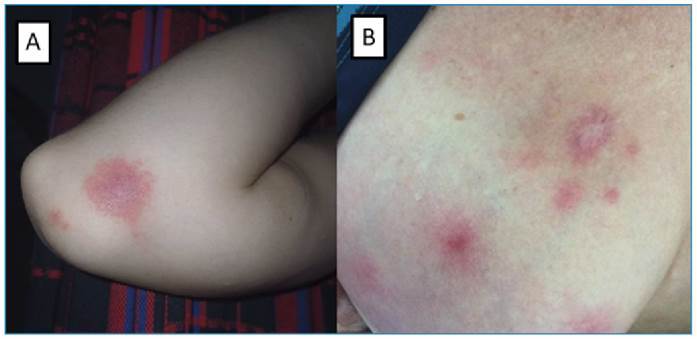
**A.** A 26-year-old female patient with a clinical presentation suggestive of DEN, including fever, rash, myalgia, asthenia, and adynamia lasting 3 days. During admission, circular erythematous lesions were noted, accompanied by a history of multiple kissing bug bites on the arm. Examination of the insect’s feces subsequently revealed the presence of *Trypanosoma cruzi*, confirming the diagnosis of acute Chagas disease. **B.** A 48-year-old female patient from a rural mountainous region in northeastern Brazil, presented with fever, upper limb arthralgia, myalgia, and a rash on the trunk and breasts. Physical examination revealed numerous erythematous lesions on the trunk, back, and breasts, some exhibiting a crusted appearance. Serological testing for Brazilian spotted fever confirmed the diagnosis by demonstrating a fourfold increase in IgG titers.

Bacterial, viral, and protozoan diseases have been implicated as mimicking or being mimicked by DEN. Infections with *Salmonella enterica* serotypes Typhi and Paratyphi, collectively known as enteric fever, present a complex clinical challenge for clinicians worldwide and are often confounded by DEN^59^ ^,^ ^62^. In a cross-sectional study of DEN patients admitted in Nepal among 95 DEN cases, typhoid fever was observed in 18 (18.95%), proving the similarities between both diseases, which may sometimes be indistinguishable^63^. Many cases of mistaken typhoid fever passing as DEN have been reported, mainly among travelers from endemic countries, including Brazil^59^ ^,^ ^63^. DEN must be considered by people traveling to endemic areas and needs to be ruled out. New *Plasmodium knowlesi* infections have also been implicated in mimicking DEN fever in some cases^64^. Cases of other viruses mimicking DEN have been described, including hantavirus, Japanese B encephalitis, and Madariaga^65^ ^-^ ^67^. Table 3 summarizes the infectious diseases that resemble DEN. Figure 2 shows the cases of acute Chagas disease and Brazilian Spotted Fever resembling DEN during the initial evaluation in the emergency room.

**TABLE 3:** Infectious diseases that can clinically resemble dengue fever.

Etiology	Infectious Diseases
**Bacterial**	Meningococcemia^7^ ^-^ ^9^, Brazilian Spotted Fever^54^, Rocky Mountain spotted fever^28^, Toxic Shock Syndrome^60^ ^,^ ^61^, Leptospirosis^51^ ^-^ ^54^, Typhoid Fever^59^ ^,^ ^62^ ^,^ ^63^, Paratyphoid Fever^59^ ^,^ ^62^ ^,^ ^63^, Endemic Typhus^63^ ^,^ ^132^, Murine Typhus^63^ ^,^ ^132^, Scrub Typhus^63^ ^,^ ^132^, Tick-borne Relapsing Fever^63^ ^,^ ^132^, Infectious Endocarditis^133^, hepatic abscess.
**Viral**	Acute HIV Infection^55^ ^,^ ^56^, EBV Infection^56^ ^,^ ^58^, CMV Infection^56^, COVID-19^19^ ^,^ ^20^ ^,^ ^22^, Influenza^21^ ^,^ ^25^, CHIKV^26^ ^,^ ^27^, ZIKV^31^ ^,^ ^32^ ^,^ ^34^, Bussuquara virus^41^ ^,^ ^42^, Cacipacoré virus^41^ ^,^ ^42^, Iguape virus^41^ ^,^ ^42^, Ilhéus virus^41^ ^,^ ^42^, Rocio virus^41^ ^,^ ^42^, Oropouche^43^ ^,^ ^46^ ^,^ ^47^ ^,^ ^135^, Mayaro^44^ ^-^ ^46^ ^,^ WNV^48^ ^-^ ^50^, Usuto virus^49^, O’nyong-nyong virus^83^ ^,^ ^84^, Sindbis virus^83^ ^,^ ^84^, Okelbo virus^49^, Barmah Forest^49^, Ross River virus^49^, HTLV-1^83^ ^,^ ^84^, Marburg, Ebola, Lassa Fever, YFV^35^ ^,^ ^36^, Rift Valley fever^49^, Saint Louis Encephalitis^49^, Measles^60^ ^,^ ^61^, Rubella^60^ ^,^ ^61^, Parvovírus B19^60^ ^,^ ^61^, Exanthema subitum (HHV-6)^60^ ^,^ ^61^, Hantavirus^65^, Tick-borne encephalitis^49^,Japanese B encephalitis^66^, Madariaga vírus^67^, Barmah Forest virus^65^ ^,^ ^67^, Hepatitis A^99^, Hepatites B^99^, Hepatites E^99^
**Other**	Malaria^14^ ^,^ ^16^ ^,^ ^64^, Acute Chagas Disease, Acute Toxoplasmosis^56^, Lyme Disease.

**HHV-6:** Human herpesvirus Type 6; **HTLV-1:** Human T-cell lymphotropic virus type I.

***Dengue and Co-infections:*** Coinfections in DEN-endemic regions present considerable diagnostic difficulties for doctors; however, targeted testing might offer confidence. Co-infections, such as DEN with malaria, chikungunya, or ZIKAV, can complicate clinical presentations owing to overlapping symptoms, including fever, thrombocytopenia, and anemia, rendering distinction challenging without specific testing^16^ ^,^ ^17^ ^,^ ^38^ ^,^ ^39^. Co-epidemics of malaria and DEN are frequently seen^17^. A recent study by Magalhaes et al., which enrolled 1,578 patients with acute fever in the Amazon region, found that 44(2.8%) patients had co-infections^17^. According to the same study, the presence of jaundice in DEN patients and spontaneous bleeding in malaria patients should raise the suspicion of coinfection. In instances of co-infection, it is crucial to vigilantly observe the indications of hemorrhage and hepatic problems, as pathogens such as malaria, chikungunya, and Zika may exacerbate the severity of DEN. Consequently, complementary diagnostic tests, such as blood smears for malaria, PCR assays for chikungunya and Zika, and NS1 antigen testing for dengue, are advised for precise diagnosis in areas with prevalent co-infections^30^ ^,^ ^36^. Cross-reactivity is a diagnostic pitfall in differentiating dengue from other diseases^30^ ^,^ ^36^ ^,^ ^38^. Cross-reactivity between DEN diagnostic tests and infections, such as chikungunya, Zika, and yellow fever, poses a significant challenge, especially in areas with multiple co-circulating infections. This overlap can lead to false-positive results, complicating clinical differentiation, and affecting both patient management and accurate epidemiological tracking. Table 4 summarizes the diagnostic methods used for each DEN mimic.

**TABLE 4:** Summary of diagnostic tools and key points for DEN mimickers.

Condition	Diagnostic Methods	Key Points
Dengue^1^ ^-^ ^3^	NS1 antigen test, RT-PCR, serology (IgM, IgG)	NS1 antigen positive in the first 5 days; PCR is useful in early stages; cross-reactivity with Zika.
Malaria^14^ ^,^ ^16^ ^,^ ^64^	Blood smear microscopy, RT-PCR, rapid diagnostic test (RDT)	Blood smear confirms Plasmodium species; RDT useful in endemic areas; PCR available in specialized labs.
Chikungunya^26^ ^,^ ^27^	PCR, serology (IgM, IgG)	PCR is preferred within the first week; serology helps in later stages, possible cross-reactivity with dengue.
Zika^31^ ^,^ ^32^ ^,^ ^34^,	PCR, serology (IgM, IgG)	PCR is essential for early diagnosis; serology may cross-react with dengue; important for pregnancy cases.
Leptospirosis^51^ ^-^ ^54^	Serology (ELISA, MAT), RT-PCR, blood culture	Microscopic agglutination test (MAT) is standard; PCR early in disease; elevated CRP in severe cases.
Typhoid Fever^59^ ^,^ ^62^ ^,^ ^63^	Blood culture, Widal test, PCR	Blood culture is gold standard; Widal test has limitations in endemic areas; PCR used for rapid diagnosis.
COVID-19^19^ ^,^ ^20^ ^,^ ^22^	PCR, antigen test, serology	PCR is primary diagnostic tool; antigen tests for early detection; serology useful for immunity assessment.
Yellow Fever^35^ ^,^ ^36^	PCR, serology (IgM, IgG), liver function tests	PCR during early infection; serology for later stages; jaundice and liver impairment suggest yellow fever.
Meningococcemia^7^ ^-^ ^9^	Blood culture, PCR, C-reactive protein (CRP)	Blood culture confirms Neisseria meningitidis; CRP elevated; immediate ceftriaxone treatment recommended.
Infectious endocarditis^133^	Blood culture, transthoracic echocardiogram, transesophageal echocardiogram	Blood cultures and ecocardiogram findings are major criteria for infectious endocarditis
Brazilian Spotted Fever^54^	Serology (ELISA, IFI), RT-PCR	An increase of at least four times in antibody titers (ELISA, IFI) between these two samples indicates a recent infection

## RHEUMATOLOGICAL CONDITIONS

***Systemic lupus erythematosus:*** Systemic lupus erythematosus (SLE) is a multisystem disease with many manifestations that may mimic infections, and are well-established triggers for the onset or exacerbation of SLE^68^ ^,^ ^69^. These conditions can frequently be confused because of their clinical similarities. DEN and SLE flare similarities include fever, arthralgia, myalgia, rash, transaminase elevation, cytopenia, commonly thrombocytopenia and lymphopenia^9^ ^,^ ^68^ ^,^ ^69^. Intriguingly, it can be so challenging to differentiate between both conditions that there are reports of SLE being misdiagnosed as DEN and vice versa, leading to delays in the correct diagnosis for both conditions^68^ ^,^ ^69^. 

In addition, the treatment strategies for these conditions differ significantly. SLE can present with severe complications, such as nephropathy and psychosis, often requiring corticosteroids and other drugs that are not typically part of the treatment regimen for DEN^70^. A matter of concern is that rare complications such as myocarditis and cardiac tamponade may occur in both conditions. However, management and treatment may differ and may be delayed^71^. Severe DEN can present with renal abnormalities, but these are less common than SLE flare^72^ ^-^ ^74^. Complement levels and anti-DNA antibodies may provide clues for SLE diagnosis, or may be triggered by DEN infection^70^ ^,^ ^71^. Renal complications associated with DEN include acute glomerulonephritis, rhabdomyolysis, and hemolytic uremic syndrome^74^. In contrast, SLE commonly manifests as lupus nephritis, primarily caused by a type III hypersensitivity reaction, resulting in the formation of immune complexes^73^ ^,^ ^75^ ^,^ ^76^.

Another difficulty arises from the fact that despite their clinical similarities, DEN serology may yield false-positive results owing to lupus autoantibodies^71^ ^,^ ^72^. Commercially available DEN IgM test kits can exhibit cross-reactivity with other flaviviruses, malaria, and leptospirosis, and may yield positive results in SLE and rheumatoid arthritis (RA)^74^ ^,^ ^77^ ^,^ ^78^. The best method for differentiation is RT-PCR tests during the initial five days of illness, which can identify DEN virus^74^ ^,^ ^75^ ^,^ ^77^. Another instrument is the DEN NS1 antigen, which can be detected with 92% sensitivity and 100% specificity within five days of DEN^76^ ^-^ ^79^. Santosa et al. proposed a diagnostic algorithm to differentiate between SLE and DEN^71^.

***Rheumatoid Arthritis:*** Historically, DEN has been recognized as a viral disease with rheumatic manifestations^80^. Before the well-established rheumatic features of CHIKV, the DEN virus had already presented with rheumatic manifestations such as polyarthralgia and myalgia, although with much less intensity than CHIKV^81^. DEN involves the muscles, tendons, joints, and bones^1^ ^-^ ^3^. Intense backache and pain in the long bones may overshadow the often-present peripheral polyarthralgia. Severe myalgia is common and creatinine phosphokinase levels are frequently elevated, which is commonly observed in DEN^79^ ^,^ ^80^. Apart from joint and bone tenderness, little is known about joints. A clue for differentiating RA from CHIKV infection is the presence of synovitis, which is usually not found in DEN^82^. 

Classical syndromes present in RA may share similarities with DEN, including fever, malaise, asthenia, myalgia, and arthritis. However, unlike DEN, which is usually an acute febrile disease^82^. Indeed, many viruses, such as DEN, may simulate the initial features of classic RA flares with fever and polyarthritis, including parvovirus B19, rubella virus, and alphaviruses such as CHIKV, o’ nyong-nyong, Mayaro, Sindbis, Okelbo, Barmah Forest, Ross River virus, hepatitis C virus, human T-cell lymphotropic virus type I, and human immunodeficiency virus^60^ ^,^ ^61^ ^,^ ^83^ ^,^ ^84^. Another significant challenge akin to SLE and other autoimmune diseases is understanding the role of viruses in triggering and exacerbating these conditions. Moreover, some studies conducted in Malaysia have indicated that DEN infection may not increase the risk of RA^85^. Other studies have reported changes in the outcomes of patients with SLE and RA with primary DEN infection^86^.

**FIGURE 3:**
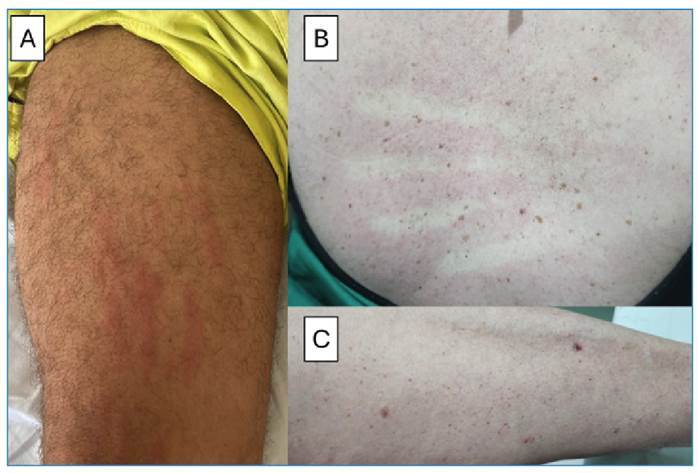
**A.** Still’s Disease with its characteristic, well-defined salmon-colored rash. **B.** Typical exanthema in DEN, demonstrating blanching under digital pressure. **C.** Petechial lesions on the patient’s arms. **Source:** *Personal archive of Dr. Jobson Lopes.*

***Still's Disease:*** Still’s disease (SD) is a rare systemic inflammatory disorder of unknown etiology characterized by the clinical triad of high-spiking fever, arthralgia, arthritis, and evanescent skin rash. The clinical presentation of SD is similar to the acute presentation of DEN^87^. When we rely on clinical signs despite laboratory alterations, the misdiagnosis of DEN is widespread^87^. SD presents as a salmon-colored skin rash, whereas DEN presents as an exanthema that disappears under digital pressure. Frequently, both types of rashes were difficult to distinguish (Figure 3)^87^. Laboratory findings are essential for the diagnosis of SD. A typical laboratory panel of a patient with SD presents with leukocytosis with neutrophilia, elevated levels of acute-phase reactants, such as CRP and ESR, elevated liver enzymes, and markedly elevated ferritin levels in the absence of rheumatoid factor (RF) and antinuclear antibodies^87^. Laboratory findings do not match the anemia, lymphopenia, and thrombocytopenia usually observed in DEN^1^ ^,^ ^2^. A conclusive diagnosis of SD is highly dependent on the physician’s judgment, as the diagnosis of SD is generally based on a thorough clinical evaluation, assessment of patient history, identification of characteristic findings, and exclusion of other possible and more common disorders^87^. 

***Dengue triggering other autoimmune diseases:*** Other rheumatological conditions characterized by autoimmunity may emerge or be mistakenly identified as DEN. The diseases discussed here may arise after an acute viral prodrome of DEN or may be misdiagnosed as DEN. An essential symptom of DEN is intense myalgia, which is sometimes the only feature associated with fever. Immune-mediated myopathies, including polymyositis, dermatomyositis, and polymyalgia rheumatica, can be misdiagnosed as DENs. Mekmangkonthong et al. reported the case of a previously healthy 9-year-old boy with sudden DEN, which evolved with high CPK levels (30, 833 mg/dL) and a generalized reduction in muscle power and weakness^88^. In this case, he hypothesized that DEN infection triggered acute necrotizing immune-mediated myopathy and ruled out juvenile dermatomyositis due to the absence of a rash^88^. 

Jayamali et al. reported another case involving a young female who developed unilateral sacroiliitis following a DEN infection^89^. However, despite this immunological possibility, it was impossible to rule out coincidental sacroiliitis co-occurring with DEN in this case. Spondylodiscitis and sacroiliitis are not commonly associated with DEN and should prompt consideration of other clinical hypotheses, including immunological trigger^89^. DEN rarely serves as a differential diagnosis, or triggers spondyloarthropathy. A cohort from Taiwan followed 12,506 newly DEN patients compared with 112,554 control patients and found increased risks of Reiter's syndrome (aHR 14.03, 95 % CI 1.63-120.58), multiple sclerosis (aHR 11.57, 95 % CI 1.8-74.4), myasthenia gravis (aHR 5.35, 95 % CI 1.43-20.02), autoimmune encephalomyelitis (aHR 3.8, 95% CI 1.85-7.8), systemic vasculitis (aHR 3.7, 95 % CI 1.11-12.28), SLE (aHR 3.5, 95% CI 1.85-6.63), and primary adrenocortical insufficiency (aHR 2.05, 95% CI 1.25-3.35)^30^. The same cohort identified five patients who developed ankylosing spondylitis in the DEN group; unfortunately, there was no difference compared with the control group^90^.

***Inflammatory Conditions and Multisystem Inflammatory Syndrome:*** Kawasaki disease (KD) is a vasculitis that primarily affects children under five years of age and often involves the coronary arteries^91^. Although the etiology remains unclear, various infections are considered potential triggers. DEN may be a significant trigger for KD^92^, particularly in endemic regions. Clinicians should consider KD when encountering prolonged fever in cases of DEN and the appearance of new clinical features such as oral changes (“strawberry tongue”), desquamation of the fingers and toes, conjunctival injection, cervical lymphadenopathy, and thrombocytosis^92^ ^,^ ^93^.

Multisystem inflammatory syndrome in children (MIS-C), an inflammatory condition associated with SARS-CoV-2 infection, presents with fever, rash, and shock, posing diagnostic challenges similar to those associated with severe DEN^94^ ^,^ ^95^. However, patients with MIS-C more frequently exhibit distinct symptoms such as conjunctival injection, oral mucosal changes, hand and foot swelling, diarrhea, and altered sensorium, whereas children with DEN more commonly present with petechiae, myalgia, headache, vomiting, bleeding, and hepatomegaly. Regarding laboratory findings, MIS-C was associated with significantly higher C-reactive protein levels. In contrast, patients with DEN typically show higher hemoglobin and hematocrit levels, lower platelet and leukocyte counts, and greater elevation in aminotransferases^95^ ^,^ ^96^. Moreover, MIS-C leads to longer hospital stays than DEN, but the duration of pediatric intensive care unit stays and mortality rates remain similar between the two diseases^97^.

## HEMATOLOGICAL CONDITIONS

***Immune thrombocytopenic purpura:*** DEN can present with numerous clinical features that may be misdiagnosed as other diseases, including thrombocytopenia. Thus, most symptomatic cases of DEN present in the viral phase with fever, myalgia, and headache, evolving into a second phase characterized by high hematocrit levels and thrombocytopenia, which usually resolves after the seventh day of illness^1^ ^-^ ^3^. During the clinical evaluation of patients with immune thrombocytopenic purpura (ITP), many physicians find low platelet levels without any symptoms despite clinical signs such as ecchymosis, gingival bleeding, or epistaxis, which may be confused with late DEN (more than four days)^97^.

The presence of isolated thrombocytopenia without lymphopenia, high hematocrit levels, and viral symptoms should raise the suspicion of ITP^97^. The absence of a history of fever is a valuable clinical indication as it represents a remarkable symptom of early DEN^1^. In contrast, DEN infection may co-occur with ITP as a comorbidity or even trigger autoimmunity^98^ ^,^ ^99^. In 2014, a fatal outcome was identified in a patient with a history of ITP who had contracted DEN due to the DEN-4 serotype and experienced hemorrhage and shock in Minas Gerais, Brazil^98^. 

Numerous reports of DEN triggering autoimmunity have been documented in the medical literature of endemic countries^90^. Identifying ITP is crucial because its treatment typically involves corticosteroids, etoposide, or other agents such as rituximab^97^. An interesting aspect of ITP and DEN is that they may be related; however, not all ITP cases are linked to DEN. Other viruses such as the Epstein-Barr virus (EBV), cytomegalovirus (CMV), human immunodeficiency virus (HIV), hepatitis B virus (HBV), hepatitis C virus (HCV), and parvovirus B19 may also be the cause^99^. The presence of thrombocytopenia and hemorrhagic symptoms alone, combined with the absence of viral syndrome, should not immediately suggest DEN, prompting further investigation and differential diagnosis.

***Thrombotic thrombocytopenic purpura:*** Another condition that may be misdiagnosed as DEN, or even related to a previous DEN virus trigger, is thrombotic thrombocytopenic purpura (TTP)^100^. Thrombotic microangiopathies (TMA) are a heterogeneous group of potentially fatal congenital and acquired diseases. TTP is the most severe form of short-term TMA, and its symptoms can mimic some of DEN's clinical features of DEN. Thrombocytopenia and neurological symptoms, such as headache and disorientation, may be misdiagnosed as DEN during an outbreak, as well as laboratory findings such as low platelet count and anemia^100^ ^-^ ^103^. 

***Myelodysplastic syndrome:*** As previously discussed, the most common blood abnormalities in DEN include increased hematocrit levels, lymphocytosis, and thrombocytopenia. Basophilia, monocytosis, and atypical lymphocytosis were found^104^. Some laboratory features of DEN may indicate a myelodysplastic syndrome (MDS). MDS consists of clonal bone marrow diseases associated with ineffective hematopoiesis, manifesting as morphological dysplasia of hematopoietic elements and peripheral cytopenias^104^. When MDS manifests with thrombocytopenia and anemia during a DEN outbreak, it may be confused with severe DEN^1^. 

***Other hematological conditions:*** Rarely, other conditions may mimic DEN. One case describes a 40-year-old white man who was a US-born engineer and had lived in Tokyo for three years. Two weeks after leaving Tokyo, he presented for evaluation of fever and diarrhea after the patient left Tokyo 2 weeks before the presentation. He spent a week at a resort in Borakai, Philippines, where he mainly relaxed on the beach and went on horseback day treks into a nearby forested area. A remarkable finding in this case was the strikingly increased population of plasma cells, plasmacytoid lymphocytes, and thrombocytopenia resembling DEN^105^. Plasmacytosis of 20% or more suggests plasma cell leukemia or myeloma and is not usually observed in DEN infection^1^ ^,^ ^105^. This patient, presenting with plasma cells and plasmacytoid lymphocytes comprising 28% of his WBC differentiation, thrombocytopenia, and constitutional symptoms, was thought clinically to have DEN fever. However, he underwent extensive evaluation to rule out the possibility of concurrent hematologic malignancy, which may be necessary in some cases of DEN with plasmacytic findings. Plasmacytosis has not frequently been reported as a feature of DEN; however, despite its rarity, it should be remembered^105^. On the other hand, a patient initially thought to have a viral infection such as DEN may have an underlying hematological malignancy, and a search for clinical clues is necessary^106^. Many reports in the literature describe a clinical picture of DEN, particularly in travelers from tropical areas where malignancies such as acute myeloid leukemia have been found^106^. Clinicians should not be influenced solely by signs, symptoms, or travel histories. These cases underscore the need to maintain a broad differential diagnosis, with complete blood count being a relevant clue in cases of leukemia and lymphoproliferative disorders^105^ ^,^ ^106^.

## OTHER CONDITIONS

***Gastrointestinal Conditions:*** As mentioned previously, DEN may present with various clinical presentations and may even be asymptomatic^1^ ^,^ ^2^ ^,^ ^107^. Abdominal symptoms such as nausea, vomiting, diarrhea, pain, right hypochondrium, and epigastric tenderness have been described^107^. Abdominal pain and painful hepatomegaly are recognized warning signs of severe DEN^107^. However, acute abdomen is less common and can be misleading for clinicians when assessing patients. Studies generally define acute abdomen as a sudden onset of abdominal pain, with fever being the primary complaint and evidence of peritonism during the examination^108^. Reports indicate that the incidence of acute abdomen in DEN is as high as 12% in Pakistan^108^. 

Patients with DEN may experience symptoms similar to those of acute surgical conditions, including acute cholecystitis, pancreatitis, appendicitis, splenic rupture, bowel perforation, gastrointestinal bleeding, and hematoma. However, DEN may also mimic an acute abdomen without any actual surgical complications such as acute pancreatitis, acute acalculous cholecystitis, non-specific peritonitis, and acute appendicitis. Acalculous cholecystitis is increasingly recognized and frequently observed in clinical practice. Although most cases can be treated without surgery, misdiagnosis and unnecessary surgical intervention can lead to poor outcomes, reinforcing the need to understand these complications^108^ ^-^ ^110^. Differential diagnosis between DEN and acute abdomen is crucial. Acute abdomens of unknown origin often require surgical intervention. Urgent surgery in a misdiagnosed case of DEN can result in iatrogenic complications and death, which can be prevented.

A complete understanding of the pathophysiological mechanisms underlying acute abdomen in DEN is still lacking. However, the virus may directly invade abdominal organs, such as the appendix, gallbladder wall, pancreas, or spleen, causing inflammation and edema. Another possible reason is a systemic inflammatory response^108^. Two hypotheses have been proposed to forward: an autoimmune response to pancreatic islet cells or direct inflammation and destruction of the pancreatic acinar by the virus^111^. Despite the lack of evidence supporting this recommendation, transabdominal ultrasonography is a useful diagnostic tool. A study was conducted on 21 patients with typical DEN and 111 with Dengue Hemorrhagic Fever (DHF). Ultrasonography was performed to detect gallbladder wall thickening, pleural effusion, and ascites. This study showed that thickening of the gallbladder wall by > 3 mm is a useful sonographic finding to confirm suspected cases of DHF^112^. Another study evaluated the association between the ultrasound assessment of gallbladder wall thickness (GBWT) and clinical outcomes among patients with DEN. This study enrolled 44 patients, most with severe DEN with GBWT (90.5% sensitivity; 69.6% specificity). GBWT had a 100% sensitivity in determining admission to critical care areas or the general ward with a specificity of 62.1%^113^.

In contrast to these studies, 160 patients with suspected DEN underwent ultrasound examinations to identify thickening of the gallbladder wall, pleural effusion, and ascites. The ultrasound findings included gallbladder wall thickening in 66 patients (41.2%)^114^. The sensitivity, specificity, and positive predictive value of ultrasound for diagnosing DEN were 58%, 84%, and 83%, respectively. The negative predictive value and accuracy were 59% and 68.8%, respectively^114^. Hence, an increased GBWT, pleural effusion, ascites, hepatomegaly, and splenomegaly strongly suggest DEN in clinically suspected cases. However, it should be noted that ultrasound should not be used as a screening tool, as negative ultrasound findings cannot rule out DEN owing to their low sensitivity^114^. DEN should be suspected in patients presenting with acute abdominal pain, particularly in endemic regions. Careful history taking, examination for DEN-related signs, serial full blood counts, DEN antigen testing, and abdominal imaging before surgical intervention may help diagnose DEN^115^. Surgery should be reserved for selected patients, and uncomplicated cases should be managed conservatively^108^. 

***Neurological Conditions:*** DEN is a neurotropic virus that causes a range of neurological syndromes affecting the central nervous system (CNS), peripheral nervous system (PNS), or both^116^. Headache is a typical symptom frequently observed in patients with DEN. Studies reveal the presence of headache in more than 97% of patients^117^. Primary headaches, such as migraine with and without aura and tension-type headaches, may be confused with DEN. The presence of fever, myalgia, and rashes should raise the suspicion of DEN, especially in endemic tropical regions^1^ ^,^ ^2^ ^,^ ^107^.

In addition, headache may present as a clinical symptom of atypical DEN. The most common neurological complications described are encephalopathy and encephalitis, with a prevalence ranging from 0.5% to 6.2%. CNS symptoms include headache, insomnia, dizziness, restlessness, altered mental status, seizures, meningitis, and muscle weakness. EEGs may show burst suppression, electrographic seizures, focal patterns, or epilepsy partialis continua. CNS symptoms may precede or follow a hemorrhage. Encephalopathy can result from hepatic failure, renal dysfunction, electrolyte abnormalities, hypoxia, or shock. DEN encephalitis can cause unconsciousness, headaches, fever, nausea, vomiting, seizures, localized neurological abnormalities, and behavioral issues^118^ ^-^ ^120^.

In DEN encephalopathy, the cerebrospinal fluid profile is typically normal. Additionally, neuroimaging studies may show diffuse cerebral edema or appear normal. Magnetic resonance imaging (MRI) is the preferred neuroimaging method; however, no specific MRI features characterize DEN encephalitis. Differential diagnoses include ADEM (acute disseminated encephalomyelitis)^118^, Japanese B encephalitis, and Chikungunya^26^ ^,^ ^27^ ^,^ ^66^. DEN encephalopathy and encephalitis should be considered when diagnosing other acute febrile encephalopathies, autoimmune encephalitis, and encephalopathy or encephalitis related to COVID-19^119^ ^,^ ^120^.

DEN can cause an ischemic or hemorrhagic stroke. Hemorrhagic strokes occur in 0.26% to 0.06% of hospitalized DEN patients. Common symptoms include fever, moderate-to-severe headache, vomiting, abrupt hemiparesis, and unconsciousness. Most individuals experience intracranial hemorrhage one week after a fever^119^ ^,^ ^120^.

Many immune-mediated syndromes can occur after DEN, including mononeuropathies, Guillain-Barré syndrome, brachial neuritis, transverse myelitis, ADEM, acute cerebellitis, opsoclonus-myoclonus syndrome, optic neuritis, and parkinsonism^118^ ^-^ ^121^. 

It is also associated with neuromuscular complications. Patients with acute flaccid quadriplegia without cranial nerve palsy or sphincteric impairment may develop hypokalemia. DEN myositis can cause muscle weakness, quadriparesis, and pulmonary failure. Owing to cytokine-mediated muscle cell injury, DEN-induced rhabdomyolysis may cause kidney damage and electrolyte problems^119^ ^,^ ^120^.

## DISCUSSION

This study is a comprehensive review of the medical literature aimed at distinguishing between DEN and other similar diagnoses and conditions in diverse scenarios.A ccurate diagnosis is crucial for effective treatment in medical practice. However, consistent execution using the most suitable methods is required. In 2024, we will experience one of the worst DEN epidemics across all states of Brazil, with over six million confirmed cases and numerous deaths by mid-year^4^ ^,^ ^5^. The potential consequences of misdiagnosis include delayed treatment of other conditions that mimic DEN, unnecessary treatments for DEN, and negative impacts on disease surveillance and control efforts^121^.

Despite the wide range of differential diagnoses, high suspicion of DEN is necessary during an epidemic, particularly in DEN-endemic areas. Laboratory methods must be available for an adequate differential diagnosis. The challenge seems to lie primarily in other arboviruses whose clinical similarities make diagnosis difficult. Studies have revealed that other arboviruses are often less suspected owing to DEN's historical impact of DEN in the country^122^. Despite the introduction of RT-qPCR Multiplex tests, which simultaneously identify DEN, Zika, and chikungunya in official laboratories during epidemics, most patients do not undergo confirmatory tests and are therefore considered to have DEN by default^122^. Serological tests are more widely available; however, cross-reactivity between viruses of the same family may reduce diagnostic certainty^30^ ^,^ ^36^. However, it is important to consider other less-discussed arboviruses in differential diagnoses in endemic areas, especially in the northern region of the country. OROV, for example, was first described in Trinidad and Tobago, with clinical characteristics resembling those of DEN. It has spread across the country, moving from the northern region to autochthonous transmission throughout Brazil^37^ ^,^ ^43^ ^-^ ^47^. In a continental country with more than 100 described arboviruses, viral surveillance, and diagnostic suspicion in cases that do not classically fit, DEN should raise the hypothesis of less common arboviruses^49^ ^,^ ^83^ ^,^ ^84^. 

Some intriguing aspects of the differential diagnosis of DEN include the wide range of diseases it encompasses, spanning almost all medical specialties. Counterintuitively, many cases of acute abdomen are initially mistaken for DEN, with some evolving into fatally^107^ ^-^ ^109^. Patients with DEN and thrombocytopenia misdiagnosed with appendicitis may develop hemorrhage and surgical complications^109^. This issue highlights the need for knowledge about DEN not only among clinical specialists but also among surgeons. A clinical study from Taiwan evaluated 328 patients with DHF/dengue shock syndrome, of whom 14 (4 men and 10 women; median age, 44 years) exhibited an acute abdomen. Presumptive diagnoses included acute cholecystitis in 10 patients, non-specific peritonitis in three patients, and acute appendicitis in one patient. These patients underwent cholecystectomy, percutaneous transhepatic gallbladder drainage, and appendectomy^123^. This study indicated that individuals with DEN who underwent surgical interventions were more prone to requiring transfusion assistance and extended hospitalization^123^. Another similar study from Sri Lanka between 2012 and 2013 described 17 cases of DEN presenting with acute abdomen. Initially, appendicitis, cholecystitis, pancreatitis, and non-specific peritonitis were suspected in eight, five, one, and three cases, respectively, highlighting the similarities among these conditions^124^. The patient with appendicitis required a blood transfusion and an extended hospital stay; four patients required ICU care, and one died^124^. Experienced surgeons in endemic areas often have one or two reports of exploratory laparotomies performed on DEN patients^123^ ^,^ ^124^. Herein, we advocate careful investigation to rule out DEN through molecular assessments in endemic areas to avoid unnecessary surgical morbidity. Serological analysis and other methods, such as the NS1 antigen test, may be necessary, as initial hematological and ultrasonographic findings can be equivocal, often creating a diagnostic and management dilemma^1^ ^,^ ^3^ ^,^ ^12^ ^,^ ^22^ ^,^ ^26^ ^,^ ^30^ ^,^ ^76^ ^-^ ^78^.

In our experience, outside of DEN epidemic periods, our infectious disease hospital frequently receives referrals for other conditions, primarily hematological and rheumatological, as well as for other infectious diseases, including sepsis. Owing to the high burden and endemicity of DEN in many areas from southeast to northeast Brazil, conditions such as ITP or even gastroenterological conditions such as cirrhosis with bacterial peritonitis are often initially managed as DEN. Similarly, bacterial conditions, such as meningococcemia, could delay antibiotic treatment, impacting therapeutic outcomes. 

In many settings, thrombocytopenia is a laboratory finding that reminds clinicians of the diagnosis of DEN. However, elevated hematocrit levels and the clinical presentation of acute febrile illness offer more significant clues. In DEN, low platelet counts typically last for approximately 5 days, after which recovery begins, with normalization occurring approximately 10 days after the onset of fever^1^ ^-^ ^3^ ^,^ ^12^. Although uncommon, fever may also be present in other thrombocytopenic conditions such as EDTA-dependent pseudothrombocytopenia, which is characterized by low platelet counts that do not respond to platelet transfusion^125^. It is important to follow the basic approach of measuring platelets using citrate and/or EDTA to avoid false thrombocytopenia and prevent diagnostic errors.

Other conditions may resemble DEN in specific situations affecting organs and systems that have not been previously mentioned. However, these usually do not account for most cases. The atypical manifestations of DEN related to myocardial involvement or arrhythmias have been well described^126^. Few cases of DEN mimicking or causing acute myocardial infarction have been reported^127^. The clinical aspects of myopericarditis may include chest pain and ST elevation on electrocardiography (EKG), often misdiagnosed as infarction in young adults^126^ ^-^ ^128^.

In 1991, the WHO Health Organization introduced the concept of syndromic management for the care of individuals with sexually transmitted infections (STIs) in developing countries. At the time, diagnostic methods were unreliable, expensive, and required sophisticated equipment and specialized training^129^. Moreover, for certain tests, patients were required to return after one or two days. This was not feasible in many locations where patients had to travel long distances to access healthcare. Even when follow-up visits occurred, the transmission period was prolonged because of delayed treatment. According to Bosu et al., syndromic management is based on identifying consistent groups of easily recognizable symptoms and signs (syndromes) and guiding appropriate measures for their control. In the context of an epidemic, there is an urgent need to recognize suspected cases and determine an appropriate therapeutic approach^129^ ^-^ ^131^. In addition, expanding laboratory testing during epidemics could help differentiate between dengue and co-circulating arboviruses such as chikungunya, Zika, and Oropouche, leading to more accurate case counts^130^ ^,^ ^131^. Thus, a syndromic approach to diseases can expedite decision-making and implementation of preventive or therapeutic measures. This can be especially important in healthcare units with lower technological complexity, such as primary healthcare (PHC).

This review raises awareness among healthcare providers regarding the challenges in accurately diagnosing DEN, especially when its complications mimic the symptoms of other diseases^132^ ^-^ ^133^. Second, this review can help differentiate DEN from other illnesses, leading to improved diagnostic protocols and a more accurate and timely identification of DEN cases, thus reducing misdiagnosis and unnecessary treatments. This can also contribute to the better utilization of healthcare resources^134^ ^-^ ^135^.

Some limitations are worth noting. First, clinical experts selected articles for this review, potentially overlooking data from the gray literature. Second, this narrative review focused on the differential diagnosis of DEN, excluding other aspects such as new diagnostic tools, prognosis, and treatment. Another limitation is the absence of a systematic article selection process. However, all experts involved in this review participated in the decision-making process regarding article inclusion and individually evaluated the relevance of articles to mitigate the risk of bias. 

## CONCLUSION

This study provides important data for clinicians and physicians who treat patients with suspected DEN in daily clinical practice. DEN presents a myriad of signs and symptoms that mimic other diseases. The differential diagnoses of DEN are broad and may involve various organs and systems. Recognizing the main differential diagnoses resembling DEN is crucial, as they can influence the determination of favorable or fatal outcomes in clinical settings. Molecular methods may be necessary to distinguish DEN from other infectious diseases. Although a DEN epidemic can increase clinical suspicion, it is essential to rule out other diseases within this context. Recognizing the main differential diagnoses resembling DEN is important as it can help decide between a favorable or fatal outcome in clinical settings. Healthcare centers in developing countries often lack laboratory facilities with sufficient technology to perform an accurate etiological diagnosis. Clinical suspicion and syndromic diagnoses are crucial during outbreaks in resource-limited settings with limited laboratory support. Emphasizing these aspects can significantly improve patient outcomes and disease management. This review aimed to assist infectious disease specialists and clinical physicians working in tropical medicine in expanding their knowledge of differential diagnoses and suspecting or confirming DEN in uncertain situations.

## References

[1] Paz-Bailey G, Adams LE, Deen J, Anderson KB, Katzelnick LC (2024). Dengue. Lancet.

[2] Roy SK, Bhattacharjee S (2021). Dengue virus: epidemiology, biology, and disease a etiology. Can J Microbiol.

[3] Castro MC, Wilson ME, Bloom DE (2017). Disease and economic burdens of dengue. Lancet Infect Dis.

[4] Gurgel-Gonçalves R, Oliveira WK, Croda J (2024). The greatest Dengue epidemic in Brazil: Surveillance, Prevention, and Control. Rev Soc Bras Med Trop.

[5] JB S, Massad E, Lobao-Neto A, Kastner R, Oliver L, Gallagher E (2022). Epidemiology and costs of dengue in Brazil: a systematic literature review. Int J Infect Dis.

[6] Ferrari R (2015). Writing narrative style literature reviews. Medical Writing.

[7] Siddiqui JA, Ameer MA, Gulick PG (2023). Meningococcemia.

[8] Gonzales Y Tucker RD, Addepalli A (2024). Fever and Rash. Emerg Med Clin North Am.

[9] Haj-Hassan TA, Thompson MJ, Mayon-White RT, Ninis N, Harnden A, Smith LF (2011). Which early 'red flag' symptoms identify children with meningococcal disease in primary care?. Br J Gen Pract.

[10] de Filippis I, Guerra Nunes PC, de Andrade CF, Gonçalves BS, de Araújo ES, Bezerra IO (2016). Fatal case of co-infection with dengue virus and Neisseria meningitidis during a dengue epidemic in the state of Rio de Janeiro, Brazil. JMM Case Rep.

[11] Souza LJ, Reis AF, de Almeida FC, Souza LA, Abukater M, Gomes MA (2008). Alteration in the erythrocyte sedimentation rate in dengue patients: analysis of 1,398 cases. Braz J Infect Dis.

[12] Anwar F, Ullah S, Aziz AUR, Rehman AU, Khan J, Tayyab M (2022). Epidemiological and hematological investigation of dengue virus infection. Microbiol Immunol.

[13] Bressan CDS, Teixeira MLB, Gouvêa MIFDS, de Pina-Costa A, Santos HFP, Calvet GA (2023). Challenges of acute febrile illness diagnosis in a national infectious diseases center in Rio de Janeiro: 16-year experience of syndromic surveillance. PLoS Negl Trop Dis.

[14] Mon NTS, Tangpukdee N, Charunwatthana P, Boonnak K, Krudsood S, Kano S (2022). Mimicking platelet indices in patients with malaria and dengue hemorrhagic fever: characteristics and clinical applications. Trop Med Health.

[15] World Health Organization (2024). Vector-borne diseases.

[16] Magalhães BM, Siqueira AM, Alexandre MA, Souza MS, Gimaque JB, Bastos MS (2014). P. vivax malaria and dengue fever co-infection: a cross-sectional study in the Brazilian Amazon. PLoS Negl Trop Dis.

[17] Ahmed A, Eldigail M, Elduma A, Breima T, Dietrich I, Ali Y (2021). First report of epidemic dengue fever and malaria co-infections among internally displaced persons in humanitarian camps of North Darfur, Sudan. Int J Infect Dis.

[18] Cardona-Arias JA, Higuita Gutiérrez LF, Carmona-Fonseca J (2023). Diagnostic Accuracy of a Thick Blood Smear Compared to qPCR for Malaria Associated with Pregnancy in Colombia. Trop Med Infect Dis.

[19] Thein TL, Ang LW, Young BE, Chen MI, Leo YS, Lye DCB (2021). Differentiating coronavirus disease 2019 (COVID-19) from influenza and dengue. Sci Rep.

[20] Gérardin P, Maillard O, Bruneau L, Accot F, Legrand F, Poubeau P (2022). Differentiating COVID-19 and dengue from other febrile illnesses in co-epidemics: Development and internal validation of COVIDENGUE scores. Travel Med Infect Dis.

[21] Wong JM, Volkman HR, Adams LE, Oliveras García C, Martinez-Quiñones A, Perez-Padilla J (2022). Clinical Features of COVID-19, Dengue, and Influenza among Adults Presenting to Emergency Departments and Urgent Care Clinics-Puerto Rico, 2012-2021. Am J Trop Med Hyg.

[22] Alla D, Alla SSM, Vempati R, Bhatt H, Sultana Q, Bhatt S (2022). Dengue & COVID-19: A Comparison and the Challenges at Hand. Cureus.

[23] Borgatta B, Pérez M, Rello J, Vidaur L, Lorente L, Socías L, pH1N1 GTEI/SEMICYUC (2012). Elevation of creatine kinase is associated with worse outcomes in 2009 pH1N1 influenza A infection. Intensive Care Med.

[24] Rosso F, Parra-Lara LG, Agudelo-Rojas OL, Martinez-Ruiz DM (2021). Differentiating Dengue from COVID-19: Comparison of Cases in Colombia. Am J Trop Med Hyg.

[25] Cunha BA, Raza M (2015). During influenza season: all influenza-like illnesses are not due to influenza: dengue mimicking influenza. J Emerg Med.

[26] Martinez JD, Garza JAC, Cuellar-Barboza A (2019). Going Viral 2019: Zika, Chikungunya, and Dengue. Dermatol Clin.

[27] Mason PJ, Haddow AJ (1957). An epidemic of virus disease in Southern Province, Tanganyika Territory, in 1952-53; an additional note on Chikungunya virus isolations and serum antibodies. Trans R Soc Trop Med Hyg.

[28] Mora JD, Licona-Enríquez JD, Álvarez-López DI, Aguilar-León DE, Álvarez-Hernández G (2021). Clinical features of patients with Rocky Mountain spotted fever, dengue and chikungunya infection. Gac Med Mex.

[29] Alvarado LI, Lorenzi OD, Torres-Velásquez BC, Sharp TM, Vargas L, Muñoz-Jordán JL (2019). Distinguishing patients with laboratory-confirmed chikungunya from dengue and other acute febrile illnesses, Puerto Rico, 2012-2015. PLoS Negl Trop Dis.

[30] Kam YH, Pok KY, Eng KE, Tan LK, Kaur S, Lee WW (2015). Sero-Prevalence and Cross-Reactivity of Chikungunya Virus Specific Anti-E2EP3 Antibodies in Arbovirus-Infected Patients. PLoS Negl Trop Dis.

[31] Lowe R, Barcellos C, Brasil P, Cruz OG, Honório NA, Kuper H (2018). The Zika Virus Epidemic in Brazil: From Discovery to Future Implications. Int J Environ Res Public Health.

[32] Sharma V, Sharma M, Dhull D, Sharma Y, Kaushik S, Kaushik S (2020). Zika virus: an emerging challenge to public health worldwide. Can J Microbiol.

[33] Mansuy JM, Lhomme S, Cazabat M, Pasquier C, Martin-Blondel G, Izopet J (2018). Detection of Zika, dengue and chikungunya viruses using single-reaction multiplex real-time RT-PCR. Diagn Microbiol Infect Dis.

[34] Matono T, Yamate R (2023). Dengue Fever with Conjunctivitis Mimicking Zika Virus Infection. Intern Med.

[35] Tuells J, Henao-Martínez AF, Franco-Paredes C (2022). Yellow Fever: A Perennial Threat. Arch Med Res.

[36] Amaku M, Coutinho FA, Massad E (2011). Why dengue and yellow fever coexist in some areas of the world and not in others?. Biosystems.

[37] Ho YL, Joelsons D, Leite GFC, Malbouisson LMS, Song ATW, Perondi B (2019). Hospital das Clínicas Yellow Fever Assistance Group. Severe yellow fever in Brazil: clinical characteristics and management. J Travel Med.

[38] Silva MMO, Tauro LB, Kikuti M, Anjos RO, Santos VC, Gonçalves TSF (2019). Concomitant Transmission of Dengue, Chikungunya, and Zika Viruses in Brazil: Clinical and Epidemiological Findings From Surveillance for Acute Febrile Illness. Clin Infect Dis.

[39] Carrillo-Hernández MY, Ruiz-Saenz J, Villamizar LJ, Gómez-Rangel SY, Martínez-Gutierrez M (2018). Co-circulation and simultaneous co-infection of dengue, chikungunya, and zika viruses in patients with febrile syndrome at the Colombian-Venezuelan border. BMC Infect Dis.

[40] Magalhaes T, Chalegre KDM, Braga C, Foy BD (2020). The Endless Challenges of Arboviral Diseases in Brazil. Trop Med Infect Dis.

[41] Figueiredo LT (2007). Emergent arboviruses in Brazil. Rev Soc Bras Med Trop.

[42] Figueiredo LT (2000). The Brazilian flaviviruses. Microbes Infect.

[43] Romero-Alvarez D, Escobar LE (2018). Oropouche fever, an emergent disease from the Americas. Microbes Infect.

[44] Acosta-Ampudia Y, Monsalve DM, Rodríguez Y, Pacheco Y, Anaya JM, Ramírez-Santana C (2018). Mayaro: an emerging viral threat?. Emerg Microbes Infect.

[45] Figueiredo ML, Figueiredo LT (2014). Emerging alphaviruses in the Americas: Chikungunya and Mayaro. Rev Soc Bras Med Trop.

[46] Lorenz C, Chiaravalloti-Neto F (2024). Brazil reports an increased incidence of oropouche and mayaro fever in the amazon region. Travel Med Infect Dis.

[47] Fujita DM, Salvador FS, da Silva Nali LH, de HF (2024). Oropouche in Brazil in 2024. J Travel Med.

[48] Rossi SL, Ross TM, Evans JD (2010). West Nile virus. Clin Lab Med.

[49] Simonin Y (2022). Usutu, West Nile, and Tick-Borne Encephalitis Viruses. Viruses.

[50] Paddock CD, Nicholson WL, Bhatnagar J, Goldsmith CS, Greer PW, Hayes EB (2006). Fatal hemorrhagic fever caused by West Nile virus in the United States. Clin Infect Dis.

[51] Jiménez JIS, Marroquin JLH, Richards GA, Amin P (2018). Leptospirosis: Report from the task force on tropical diseases by the World Federation of Societies of Intensive and Critical Care Medicine. J Crit Care.

[52] Kartick C, Bharathi GSJ, Surya P, Anwesh M, Arun S, Muruganandam N (2017). Outbreak investigation of fever mimicking dengue in Havelock Island, an important tourist destination in the Andaman & Nicobar Archipelago, 2014. Epidemiol Infect.

[53] Fornazari F, Richini-Pereira VB, Joaquim SF, Nachtigall PG, Langoni H (2021). Leptospirosis diagnosis among patients suspected of dengue fever in Brazil. J Venom Anim Toxins Incl Trop Dis.

[54] Maillard O, Hirschinger D, Bénéteau S, Koumar Y, Vague A, Girerd R (2023). C-reactive protein: An easy marker for early differentiation between leptospirosis and dengue fever in endemic area. PLoS One.

[55] Matsuda EM, Colpas DR, Campos NC, Coelho LP, Carmo AM, Brígido LF (2017). Undiagnosed acute HIV infection identified through RNA testing of pooled serum samples obtained during a dengue outbreak in São Paulo, Brazil. Rev Soc Bras Med Trop.

[56] Senaratne UTN, Murugananthan K, Sirisena PDNN, Carr JM, Noordeen F (2020). Dengue virus co-infections with multiple serotypes do not result in a different clinical outcome compared to mono-infections. Epidemiol Infect.

[57] Sylvester JE, Buchanan BK, Silva TW (2023). Infectious Mononucleosis: Rapid Evidence Review. Am Fam Physician.

[58] Boyd K, Harrison JM, Kavanaugh MJ (2018). False-Positive Monospot in a Returning Traveler with Dengue Fever. Mil Med.

[59] Cunha BA, Johnson D, McDermott B (2009). Atypical dengue fever mimicking typhoid fever in a college student traveler. Am J Med.

[60] Da Silva Carneiro SC, Cestari T, Allen SH, Ramos e-Silva M (2007). Viral exanthems in the tropics. Clinics in Dermatology.

[61] Palhares D (2021). Exanthematic dengue fever mimicking rubella. An Bras Dermatol.

[62] Mahato AK, Shrestha N, Gharti SB, Shah M (2022). Typhoid Fever among Patients Diagnosed with Dengue in a Tertiary Care Centre: A Descriptive Cross-sectional Study. J Nepal Med Assoc.

[63] Tan L, Beersma TM, van Beek Y, van Genderen PJ (2011). Two travellers suffering from typhus. Ned Tijdschr Geneeskd.

[64] Azira NM, Zairi NZ, Amry AR, Zeehaida M (2012). Case series of naturally acquired Plasmodium knowlesi infection in a tertiary teaching hospital. Trop Biomed.

[65] Hamidon BB, Saadiah S (2003). Seoul hantavirus infection mimicking dengue fever. Med J Malaysia.

[66] Sarkar R, Paul R, Thakur I, Ghosh R, Singh S, Mani A (2019). Encephalitis Due to Dengue Virus Infection Mimicking Japanese B Encephalitis: Two Case Reports. J Assoc Physicians India.

[67] Blohm GM, Lednicky JA, White SK, Mavian CN, Márquez MC, González-García KP (2018). Madariaga Virus: Identification of a Lineage III Strain in a Venezuelan Child With Acute Undifferentiated Febrile Illness, in the Setting of a Possible Equine Epizootic. Clin Infect Dis.

[68] de Souza SP, de Moura CG (2010). Dengue mimicking a lupus flare. J Clin Rheumatol.

[69] Sh Talib, Bhattu S, Bhattu R, Deshpande S, Dahiphale D (2013). Dengue fever triggering systemic lupus erythematosus and lupus nephritis: a case report. Int Med Case Rep J.

[70] Bernal C, Acosta Colmán I, Cardozo F, Waggoner JJ, Cantero C, Acosta ME (2021). Delayed Diagnosis of Dengue in a Patient With Systemic Lupus Erythematosus. J Clin Rheumatol.

[71] Santosa A, Poh Z, Teng GG (2012). Delayed diagnosis of systemic lupus erythematosus due to misinterpretation of dengue serology. Scand J Rheumatol.

[72] Zandman-Goddard G, Schoenfeld Y (2005). Infections and SLE. Autoimmunity.

[73] Kumar S, Iuga A, Jean R (2010). Cardiac tamponade in a patient with dengue fever and lupus nephritis: a case report. J Intensive Care Med.

[74] Gurugama P, Jayarajah U, Wanigasuriya K, Wijewickrama A, Perera J, Seneviratne SL (2018). Renal manifestations of dengue virus infections. J Clin Virol.

[75] Musa R, Brent LH, Qurie A (2023). Lupus Nephritis.

[76] Eswarappa M, Reddy SB, John MM, Suryadevara S, Madhyashatha RP (2019). Renal manifestations of dengue viral infection. Saudi J Kidney Dis Transpl.

[77] Hunsperger EA, Yoksan S, Buchy P, Nguyen VC, Sekaran SD, Enria DA (2009). Evaluation of commercially available antidengue virus immunoglobulin M tests. Emerg Infect Dis.

[78] Tian R, Yan H, Jiang Y, Wu A, Li L, Yang Z (2022). Detection and typing of dengue virus by one-step RT-PCR-based high-resolution melting assay. Virus Genes.

[79] Muller DA, Depelsenaire AC, Young PR (2017). Clinical and Laboratory Diagnosis of Dengue Virus Infection. J Infect Dis.

[80] Adebajo AO (1996). Dengue arthritis. Br J Rheumatol.

[81] Santos LLM, de Aquino EC, Fernandes SM, Ternes YMF, Feres VCR (2023). Dengue, chikungunya, and Zika virus infections in Latin America and the Caribbean: a systematic review. Rev Panam Salud Publica.

[82] Amaral JK, Bilsborrow JB, Schoen RT (2020). Chronic Chikungunya Arthritis and Rheumatoid Arthritis: What They Have in Common. Am J Med.

[83] Perl A (1999). Mechanisms of viral pathogenesis in rheumatic disease. Ann Rheum Dis.

[84] Marks M, Marks JL (2016). Viral arthritis. Clin Med.

[85] Tan LK, Too CL, Nurul-Aain AF, Siti-Aisyah AA, Sulaiman W, Osman A (2021). OP0096 Exposure to Dengue Infection Does Not Raise Risk of Rheumatoid Arthritis: Findings From the Malaysian Epidemiological Investigation of Rheumatoid Arthritis (MyEIRA) Case-Control Study. Ann Rheum Dis.

[86] de Abreu MM, Maiorano AC, Tedeschi SK, Yoshida K, Lin TC, Solomon DH (2018). Outcomes of lupus and rheumatoid arthritis patients with primary dengue infection: A seven-year report from Brazil. Semin Arthritis Rheum.

[87] Efthimiou P, Kontzias A, Hur P, Rodha K, Ramakrishna GS, Nakasato P (2021). Adult-onset Still's disease in focus: Clinical manifestations, diagnosis, treatment, and unmet needs in the era of targeted therapies. Semin Arthritis Rheum.

[88] Mekmangkonthong A, Amornvit J, Numkarunarunrote N, Veeravigrom M, Khaosut P (2022). Dengue infection triggered immune mediated necrotizing myopathy in children: a case report and literature review. Pediatr Rheumatol Online J.

[89] Jayamali WD, Herath HMMTB, Kulatunga A (2017). A young female presenting with unilateral sacroiliitis following dengue virus infection: a case report. J Med Case Rep.

[90] Li HM, Huang YK, Su YC, Kao CH (2018). Increased risk of autoimmune diseases in dengue patients: A population-based cohort study. J Infect.

[91] Aggarwal R, Pilania RK, Sharma S, Kumar A, Dhaliwal M, Rawat A (2023). Kawasaki disease and the environment: an enigmatic interplay. Front Immunol.

[92] Jagadeesh A, Krishnamurthy S, Mahadevan S (2016). Kawasaki Disease in a 2-year-old Child with Dengue Fever. Indian J Pediatr.

[93] Guleria S, Jindal AK, Pandiarajan V, Singh MP, Singh S (2018). Dengue-Triggered Kawasaki Disease: A Report of 2 Cases. J Clin Rheumatol.

[94] Samprathi M, Narayanappa S, Sridhar M, Ramachandra P, Vemgal P (2021). Multisystem Inflammatory Syndrome in Children: A Mimicker of Severe Dengue. Indian J Pediatr.

[95] Dhooria GS, Kakkar S, Pooni PA, Bhat D, Bhargava S, Arora K (2021). Comparison of Clinical Features and Outcome of Dengue Fever and Multisystem Inflammatory Syndrome in Children Associated With COVID-19 (MIS-C). Indian Pediatr.

[96] Randhawa MS, Angurana SK, Nallasamy K, Kumar M, Ravikumar N, Awasthi P (2023). Comparison of Multisystem Inflammatory Syndrome (MIS-C) and Dengue in Hospitalized Children. Indian J Pediatr.

[97] De Souza S, Angelini D (2021). Updated guidelines for immune thrombocytopenic purpura: Expanded management options. Cleve Clin J Med.

[98] Amâncio FF, Pereira MA, Iani FC, D'anunciação L, de Almeida JL, Soares JA (2014). Fatal outcome of infection by dengue 4 in a patient with thrombocytopenic purpura as a comorbid condition in Brazil. Rev Inst Med Trop Sao Paulo.

[99] Ramírez-Fonseca T, Segarra-Torres A, Jaume-Anselmi F, Ramírez-Rivera J (2015). Dengue Fever: A Rare Cause Of Immune Thrombocytopenia. Bol Asoc Med P R.

[100] Rana A, Ahlawat P, Upadhyay P, Gupta A, Bansal A (2023). Dengue Infection Triggering Concurrent Thrombotic Thrombocytopenic Purpura in a Case of Chronic Idiopathic Thrombocytopenic Purpura. Cureus.

[101] Kumar S, Khadwal A, Verma S, Singhi SC (2013). Immune thrombocytopenic purpura due to mixed viral infections. Indian J Pediatr.

[102] Bastos MLA, Araújo RMO, Oliveira DS, Cavalcante ANM, Silva GBD (2018). Thrombotic thrombocytopenic purpura associated with dengue and chikungunya virus coinfection: case report during an epidemic period. Rev Inst Med Trop Sao Paulo.

[103] Gogireddy RR, Kumar V, Ranjit S, Natraj R, Venkatachalapathy P, Jayakumar I (2020). Thrombotic thrombocytopenic purpura in a 2.5-year-old boy with dengue infection: a rare complication. Paediatr Int Child Health.

[104] Cazzola M (2020). Myelodysplastic Syndromes. N Engl J Med.

[105] Gawoski JM, Ooi WW (2003). Dengue fever mimicking plasma cell leukemia. Arch Pathol Lab Med.

[106] Yapor L, Zahid M, Shrestha N, Walck R, Schreiber Z, Adrish M (2020). Case report: An unusual presentation of acute promyelocytic leukemia in a middle aged female mimicking dengue infection. Medicine.

[107] Ministério da Saúde (MS). Secretaria de Vigilância em Saúde. Departamento de Vigilância das Doenças Transmissíveis - Dengue (2024). Diagnóstico e Manejo clínico: Adulto e criança.

[108] Jayarajah U, Lahiru M, De Zoysa I, Seneviratne SL (2021). Dengue Infections and the Surgical Patient. Am J Trop Med Hyg.

[109] McFarlane ME, Plummer JM, Leake PA, Powell L, Chand V, Chung S (2013). Dengue fever mimicking acute appendicitis: A case report. Int J Surg Case Rep.

[110] Senanayake MP, Samarasinghe M (2014). Acute appendicitis complicated by mass formation occurring simultaneously with serologically proven dengue fever: a case report. J Med Case Rep.

[111] Correa R, Ortega-Loubon C, Zapata-Castro LE, Armién B, Culquichicón C (2019). Dengue with Hemorrhagic Manifestations and Acute Pancreatitis: Case Report and Review. Cureus.

[112] Quiroz-Moreno R, Méndez GF, Ovando-Rivera KM (2006). Utilidad clínica del ultrasonido en la identificación de dengue hromorrágico [Clinical utility of ultrasound in the identification of dengue hemorrhagic fever]. Rev Med Inst Mex Seguro Soc.

[113] Ibrahim MA, Hamzah SS, Md Noor J, Mohamad MIK, Mokhtar MF, Isa MR (2022). The association of ultrasound assessment of gallbladder wall thickness with dengue fever severity. Ultrasound J.

[114] Parmar J, Mohan C, Kumar GP, Vora M (2017). Ultrasound is Not Useful as a Screening Tool for Dengue Fever. Pol J Radiol.

[115] Premaratna R, Bailey MS, Ratnasena BG, de Silva HJ (2007). Dengue fever mimicking acute appendicitis. Trans R Soc Trop Med Hyg.

[116] Prabhat N, Ray S, Chakravarty K, Kathuria H, Saravana S, Singh D (2020). Atypical neurological manifestations of dengue fever: a case series and mini review. Postgrad Med J.

[117] Kulkarni R, Pujari S, Gupta D (2021). Neurological Manifestations of Dengue Fever. Ann Indian Acad Neurol.

[118] Diallo A, Dembele Y, Michaud C, Jean M, Niang M, Meliani P (2020). Acute disseminated encephalomyelitis after dengue. IDCases.

[119] Trivedi S, Chakravarty A (2022). Neurological Complications of Dengue Fever. Curr Neurol Neurosci Rep.

[120] Verma R, Sahu R, Holla V (2014). Neurological manifestations of dengue infection: a review. J Neurol Sci.

[121] JB S, Massad E, Lobao-Neto A, Kastner R, Oliver L, Gallagher E (2022). Epidemiology and costs of dengue in Brazil: a systematic literature review. Int J Infect Dis.

[122] Ribas Freitas AR, Pinheiro Chagas AA, Siqueira AM, Pamplona de Góes Cavalcanti L (2024). How much of the current serious arbovirus epidemic in Brazil is dengue and how much is chikungunya?. Lancet Reg Health Am.

[123] Khor BS, Liu JW, Lee IK, Yang KD (2006). Dengue hemorrhagic fever patients with acute abdomen: clinical experience of 14 cases. Am J Trop Med Hyg.

[124] Jayasundara B, Perera L, de Silva A (2017). Dengue fever may mislead the surgeons when it presents as an acute abdomen. Asian Pac J Trop Med.

[125] Shrestha GS, Basnet B, Nepal G, Lamichhane R, Gaire P, Shrestha R (2021). EDTA-dependent pseudo thrombocytopenia mimicking dengue fever-associated persistent thrombocytopenia: A case report. Clin Case Rep.

[126] Araiza-Garaygordobil D, García-Martínez CE, Burgos LM, Saldarriaga C, Liblik K, Mendoza I (2021). Neglected Tropical Diseases and other Infectious Diseases affecting the Heart (the NET-Heart) project. Dengue and the heart. Cardiovasc J Afr.

[127] Ramanathan K, Teo L, Raymond WC, MacLaren G (2015). Dengue Myopericarditis Mimicking Acute Myocardial Infarction. Circulation.

[128] Farias LABG, Beserra FLCN, Fernandes L, Teixeira AAR, Ferragut JM, Girão ES (2019). Myocarditis Following Recent Chikungunya and Dengue Virus Coinfection: A Case Report. Arq Bras Cardiol.

[129] Bosu WK (1999). Syndromic management of sexually transmitted diseases: is it rational or scientific?. Trop Med Int Health.

[130] Yang X, Quam MBM, Zhang T, Sang S (2021). Global burden for dengue and the evolving pattern in the past 30 years. J Travel Med.

[131] Lenharo M (2024). Brazil's record dengue surge: why a vaccine campaign is unlikely to stop it. Nature.

[132] Amritha J, Raveenthiran V (2022). Concurrent Scrub Typhus and Dengue Fever Mimicking Acute Appendicitis. Indian Pediatr.

[133] Alemad SA, Halboup AM, Aladeeb K, Al-Saleh M, Al-Kufiley N (2021). Coinfection with dengue and hepatitis A complicated with infective endocarditis in a Yemeni patient: a case report. J Med Case Rep.

[134] Ribas Freitas AR, Lima AS, Rodrigues R, Alves de Oliveira E, Andrade JS, Cavalcanti LPG (2024). Excess mortality associated with chikungunya epidemic in Southeast Brazil, 2023. Front Trop Dis.

[135] Garcia-filho C, Lima-Neto AS, Maia AMPC, Silva LOR, Monteiro HS, Marques KCA (2024). A Case of Vertical Transmission of Oropouche Virus in Brazil. N Engl J Med.

